# Heterogeneous multimodal biomarkers analysis for Alzheimer’s disease via Bayesian network

**DOI:** 10.1186/s13637-016-0046-9

**Published:** 2016-08-19

**Authors:** Yan Jin, Yi Su, Xiao-Hua Zhou, Shuai Huang, The Alzheimer’s Disease Neuroimaging Initiative

**Affiliations:** 1 Industrial Engineering Department, University of Washington, Seattle, WA USA; 2 Department of Radiology, Washington University in St. Louis, St. Louis, MO USA; 3 Department of Biostatistics, University of Washington, Seattle, WA USA

**Keywords:** Alzheimer’s disease, Bayesian network, Multimodal biomarkers, Heterogeneous, ADNI

## Abstract

By 2050, it is estimated that the number of worldwide Alzheimer’s disease (AD) patients will quadruple from the current number of 36 million, while no proven disease-modifying treatments are available. At present, the underlying disease mechanisms remain under investigation, and recent studies suggest that the disease involves multiple etiological pathways. To better understand the disease and develop treatment strategies, a number of ongoing studies including the Alzheimer’s Disease Neuroimaging Initiative (ADNI) enroll many study participants and acquire a large number of biomarkers from various modalities including demographic, genotyping, fluid biomarkers, neuroimaging, neuropsychometric test, and clinical assessments. However, a systematic approach that can integrate all the collected data is lacking. The overarching goal of our study is to use machine learning techniques to understand the relationships among different biomarkers and to establish a system-level model that can better describe the interactions among biomarkers and provide superior diagnostic and prognostic information. In this pilot study, we use Bayesian network (BN) to analyze multimodal data from ADNI, including demographics, volumetric MRI, PET, genotypes, and neuropsychometric measurements and demonstrate our approach to have superior prediction accuracy.

## Introduction

Alzheimer’s disease (AD) is a highly prevalent neurodegenrative disease and is widely recognized as a major, escalating epidemic and a worldwide challenge to global health care systems [1]. Considerable research efforts have been devoted to establish a disease model of AD that could lead to greater understanding of the events that occur in AD. One major development is the A *β* hypothesis that assumes AD begins with abnormal processing of transmembrane A *β* precursor protein (APP). Such a malfunction of the APP metabolism will in turn trigger a series of pathological events, resulting in the toxic beta-amyloid plaque in the human brain which is one defining characteristic of AD.

This disease model has been articulated in Jack et al. [2] who presented a hypothetical model for biomarker dynamics in AD pathogenesis. The model begins with the abnormal deposition of A *β* fibrils, as evidenced by a corresponding drop in the levels of soluble A *β*42 in cerebrospinal fluid (CSF) and increased retention of the amyloid positron emission tomography (PET) radioactive tracers in the cortex. Subsequently, neurodegeneration and synaptic dysfunction follows, indicated by increased levels of CSF tau protein, brain atrophy, and decreased glucose metabolism measured using [^18^F]-fluorodeoxyglucose (FDG) PET. As neuronal degeneration progresses, atrophy in certain areas typical of AD such as the hippocampus regions becomes detectable by magnetic resonance imaging (MRI). So far, Jack’s model has been widely studied, confirmed, refined, and enriched. While many details in the disease model are still unknown, investigators from academia and the pharmaceutical industry have been actively developing biomarkers to gain better and more accurate knowledge of the mechanisms of AD pathogenesis and progression to facilitate a range of clinical tasks such as early diagnosis, treatment efficacy evaluation, treatment planning, better clinical trial design, and drug developments.

While most of the existing efforts mentioned above focus on single modality of biomarkers, recently, there have been a few studies that proposed to study many biomarkers of heterogeneous nature jointly. For instance, Ye et al. [3] integrated multiple complementary data and initiated the work to use the multiple kernel learning method for multimodal integration for AD research. Zhang et al. did and regression [5] based on multimodality data and achieved better prediction accuracy than those models with a single biomarker. However, most of these works focus on prediction. Less effort has been devoted to study the interactions of these multimodal biomakers for better understanding of the disease as a whole.

Thus, in our study, we take a systematical perspective to study patterns of disease progression. We take into consideration multimodal biomarkers such as APOE (apolipoprotein E) genotypes, SNP variants, demographics, FDG-PET, amyloid PET, MRI, and neuropsychological assessment. We adopt a powerful machine learning model, the Bayesian Network (BN), as the major tool for studying the influential relationships among the variables. A main premise of using BN model for multimodal biomarker integration is that it could provide more details regarding the potential mechanism of the disease progression than those black-box prediction models [3–5]. Specifically, while the existing black-box prediction models throw in all the multimodal biomarkers as predictors parallel in the prediction equation regardless of their heterogeneous clinical nature, their clinical roles are not revealed since each biomarker is assigned with a quantitative weight in the prediction equation that only determines whether or not the biomarker is important. Moreover, this weight is not an absolute presentation of evidence, as it is essentially a multivariate concept that depends on the existence of other biomarkers in the equation. This results in the risk of excluding important biomarkers which hold significant clinical value but not significant statistical prediction value due to redundancy with other biomarkers. Also, from these black-box prediction models, there is no indication of how the biomarkers influence each other, whether or not some biomarkers mediate the effects from other biomarkers to disease outcomes. Presumably, the relationships between the multimodal biomarkers could be very complex, and our study is motivated by the lack of capacity of existing multimodal biomarker integration methods to discover and model these relationships. On the other hand, although not a causal model, BN models have been found very effective in a range of applications to study the “layers” of influence among variables. It could lead to very useful knowledge regarding the “chain reaction” of a sequence of events captured by the biomarkers’ measurements. BN is a powerful data-driven model that seeks the best mechanistic model that is consistent with a set of measurements from a cohort of patients. Thus, it translates naturally into a semantic description of the disease similar to a clinician’s intuitive description of its progression.

The remainder of the paper is structured as follows: In Section 2, we will provide description of the dataset that will be used in this study and the BN, particularly the mixed type Bayesian network due to the heterogeneous nature of the biomarkers. In Section 3, we will present the learning results and validation efforts. We then conclude our study in Section 4.

## Methods

### Data

The data used in this paper were obtained from ADNI database adni.loni.usc.edu. The primary goal of ADNI has been to test whether the serial MRI, PET, other biological markers, and clinical and neuropsychological assessment can be combined to measure the progression of mild cognitive impairment (MCI) and early AD. Determination of sensitive and specific markers of very early AD progression is intended to aid researchers and clinicians to develop new treatments and monitor their effectiveness, as well as lessen the time and cost of clinical trials.

ADNI is the result of efforts of many co-investigators from academic institutions and private corporations, subjects have been recruited from over 50 sites across the USA and Canada. The initial goal of ADNI was to recruit 800 adults, aged 55 to 90, to participate in the research with approximately 200 cognitively normal older individuals followed up for 3 years, 400 people with MCI followed up for 3 years, and 200 people with early AD followed up for 2 years.

### Subjects

The ADNI general eligibility criteria are described at www.adni-info.org. Briefly, subjects are between 55 and 90 years of age, having a study partner able to provide an independent evaluation of functioning. Specific psychoactive medications will be excluded. The general inclusion/exclusion criteria are as follows: (1) healthy subjects: mini-mental state examination (MMSE) scores between 24 and 30, a Clinical Dementia Rating (CDR) of 0, non-depressed, non-MCI, and non-demented; (2) MCI subjects: MMSE scores between 24 and 30, a memory complaint, having objective memory loss measured by education adjusted scores on Wechsler Memory Scale Logical Memory II, a CDR of 0.5, absence of significant levels of impairment in other cognitive domains, essentially preserved activities of daily living, and an absence of dementia; and (3) mild AD: MMSE scores between 20 and 26, CDR of 0.5 or 1.0, and meets the National Institute of Neurological and Communicative Disorders and Stroke and the Alzheimer’s Disease and Related Disorders Association (NINCDS/ADRDA) criteria for probable AD.

Our study includes the baseline measurements of 517 ADNI subjects. The cohort contains 114 AD patients, 283 MCI patients, and 120 healthy controls. Table 1 lists the demographics of these subjects.

**Table 1:** Subject information at baseline

	AD (*n*=114; 47 M/67 F)			MCI (*n*=283; 128 M/155 F)			HC (*n*=120; 52 M/68 F)		
	Mean	SD	Range	Mean	SD	Range	Mean	SD	Range
Age	74.6	8.1	56.5–89.6	73.9	6.7	58.5–90.6	73.4	7.3	55.0–89.6
Edu	16.0	2.6	8.0–20.0	16.4	2.7	9.0–20.0	16.8	2.6	9.0–20.0
MMSE	23.8	1.6	20.0–26.0	27.0	2.1	24.0–30.0	28.8	1.9	24.0–30.0
ADAS	15.5	7.8	4.0–51.0	14.6	9.5	0.0–51.0	10.8	8.8	3.0–31.0

### Biomarkers

The description about biomarkers to be analyzed is listed in Table 2. These biomarkers are heterogeneous in terms of both clinical nature and statistical characteristics. While this list is still limited, it provides a good presentation of the genetic, demographic, neuroimaging, and clinical aspects of the disease. Among these markers, some are categorical biomarkers, such as sex (male or female) and SNPs (carrier or non-carrier), while some are numeric biomarkers such as some clinical measurements. Note that we also include some SNPs variants which are the top genetic risk factors for AD reported at http://www.alzgene.org/TopResults.asp.

**Table 2:** Description of heterogeneous multimodal biomarkers

Biomarker	Description
Age	Age
Sex	Gender
Edu	Years of education
FDG	Average FDG-PET
AV45	Average AV45 SUVR
HippoNV	The normalized hippocampus volume
APOE4	Apolipoprotein E4 polymorphism
rs3818361	CR1 gene rs3818361 polymorphism
rs744373	BIN1 gene rs744373 polymorphism
rs11136000	Clusterin CLU gene rs11136000 polymorphism
rs610932	MS4A6A gene rs610932 polymorphism
rs3851179	PICALM gene rs3851179 polymorphism
rs3764650	ABCA7 gene rs3764650 polymorphism
rs3865444	CD33 gene rs3865444 polymorphism
MMSE	Mini-mental state examination
ADAS-cog	Alzheimer’s Disease Assessment Scale

### Bayesian network

A BN is a graphical model that characterizes the influential relationships among variables *X*={*X*_*v*_;*v*∈*V*}. Let *D*=(*V*,*E*) be a directed acyclic graph (DAG), where *V* is a finite set of nodes and *E* is a finite set of directed edges between the nodes. The DAG defines the structure of the BN. Each node *v*∈*V* in the graph corresponds to a random variable *X*_*v*_, i.e., in our study, a biomarker is a variable. In the DAG, the relationship between each variable *X*_*v*_ with its parent variables denoted as pa(*v*) can be characterized as a conditional probability distribution, *p*(*x*_*v*_|*x*_pa(*v*)_). Then, the joint probability distribution of a BN could be deduced as

1 $$  p(x)=\prod\limits_{v \in V}p(x_{v} | x_{pa(v)})  $$

For this reason, the set of conditional probability distributions for all variables in the network, denoted as $\mathcal {P}$, is called the parameter of the BN. A Bayesian network for a set of random variables *X* is then the pair $(D,\mathcal {P})$.

### Mixed type Bayesian network

In this paper, we adopt the mixed type Bayesian network model that handles both discrete and continuous variables, which is developed in [6]. For mixed type BNs, the set of nodes *V* can be further specified as *V*=*Δ*∪T, where *Δ* and T are the sets of discrete and continuous nodes, respectively. The set of variables *X* can then be denoted as *X*={*X*_*v*_;*v*∈*V*}=(*I*,*Y*)={(*I*_*δ*_,*Y*_*τ*_);*δ*∈*Δ*,*τ*∈T}, where *I* and *Y* are the sets of discrete and continuous variables, respectively. For a discrete variable *δ*, we let $\mathcal {I}_{\sigma }$ denote the set of levels.

It has been a challenge to model the mixed type Bayesian network. As mentioned earlier, a BN consists of the structure *D* and the parameter $\mathcal {P}$. The central challenge for modeling mixed type Bayesian network is the development of appropriate models for characterizing $\mathcal {P}$. In our study, we follow the seminar work in [6] that models the joint probability distribution by factorizing it into a discrete part and a mixed part, so

2 $$  p(x)=p(i,y)=\prod\limits_{\delta \in \Delta}p\left(i_{\delta}|i_{\text{pa}(\delta)}\right)\cdot\prod\limits_{\tau \in \mathrm{T}}p\left(y_{\tau}|i_{\text{pa}(\tau)},y_{\text{pa}(\tau)}\right)  $$

where the first part of products of conditional probabilities is for discrete nodes and the second part is for continuous nodes.

For discrete nodes, conditional probabilities are parameterized as

3 $$  \theta_{i_{\sigma}|i_{\text{pa}(\sigma)}}=p\left(i_{\sigma} | i_{\text{pa}(\sigma)},\theta_{\sigma|i_{\text{pa}(\sigma)}}\right),  $$

where $\theta _{\sigma |i_{\text {pa}(\sigma)}}=(\theta _{i_{\sigma }|i_{\text {pa}(\sigma)}})_{i_{\sigma } \in \mathcal {I}_{\sigma }}$. The parameters are subject to the constraints that $\sum _{i_{\sigma } \in \mathcal {I}_{\sigma }}\theta _{i_{\sigma }|i_{\text {pa}(\sigma)}}=1$ and $0\leq \theta _{i_{\sigma }|i_{\text {pa}(\sigma)}}\leq 1$.

For continuous nodes, the local probability distributions are Gaussian linear regressions on the continuous parents with parameters depending on the configuration of the discrete parents, as shown in below:

4 $$  \theta_{\tau|i_{\text{pa}(\tau)}}=\left(b_{\tau|i_{\text{pa}(\tau)}}, w_{\tau|i_{\text{pa}(\tau)}}, \sigma_{\tau|i_{\text{pa}(\tau)}}^{2}\right),  $$

so that

$$\begin{array}{*{20}l} & Y_{\tau}|i_{\text{pa}(\tau)},y_{\text{pa}(\tau)},\theta_{\tau|i_{\text{pa}(\tau)}} \\ \simeq & \mathcal{N}\left(b_{\tau|i_{\text{pa}(\tau)}}+ y_{\text{pa}(\tau)}w_{\tau|i_{\text{pa}(\tau)}}, \sigma_{\tau|i_{\text{pa}(\tau)}}^{2}\right). \end{array} $$

### Learning of mixed type BN from data

With the BN model specified for mixed type variables, the next task is to identify a structure learning algorithm that can find the optimal DAG structure. The basic formulation of this problem, according to the score-based method, starts with a dataset *T* and a scoring function *ϕ*. Then, the task is to find a Bayesian network $B \in \mathcal {B}_{n}$ that maximizes the values *ϕ*(*B*,*T*). The standard methodology is to use search algorithms, such as heuristic search, greedy hill-climbing, genetic algorithms, and tabu search, conducted over eligible search space $\mathcal {B}_{n}$ to search the DAG structure that maximizes the score. In this study, we use the score function developed in [7] for mixed type BN, which can be readily implemented in the R package “bnlearn” [7]. After having identified the optimal DAG structure, parameter estimation could be conducted via maximum likelihood estimation according to (2). We refer interested readers to [8–10] for more details of the learning algorithms for mixed type BN.

## Results

We apply the mixted type BN on the heterogeneous biomarkers of the ADNI cohort we have collected. In order to identify a stable DAG structure, first, we use a bootstrap method to generate 100 new training sets by sampling the original data set with replacement, then, learn the optimal DAG structure on each bootstrapped dataset. We then derive the final DAG structure by keeping those arcs which appear at least in half of these DAG structures learned from bootstrapped datasets. This strategy has been suggested in previous works for BN applications [11] that has been found effective to robustify the learning result. Note that, here, we also utilize the prior knowledge in the learning of the DAG structure, i.e., the genetic factors could be parents of other factors not the other way around, while the disease outcome variables such as ADAS-cog and MMSE score could only be in the bottom of the BN model. This prior knowledge is used in the BN learning and greatly reduces the search space of the eligible DAG structures. Note that, to impute missing values, the median is used for continuous variables while the mode is used for discrete variable.

**Fig. 1:**
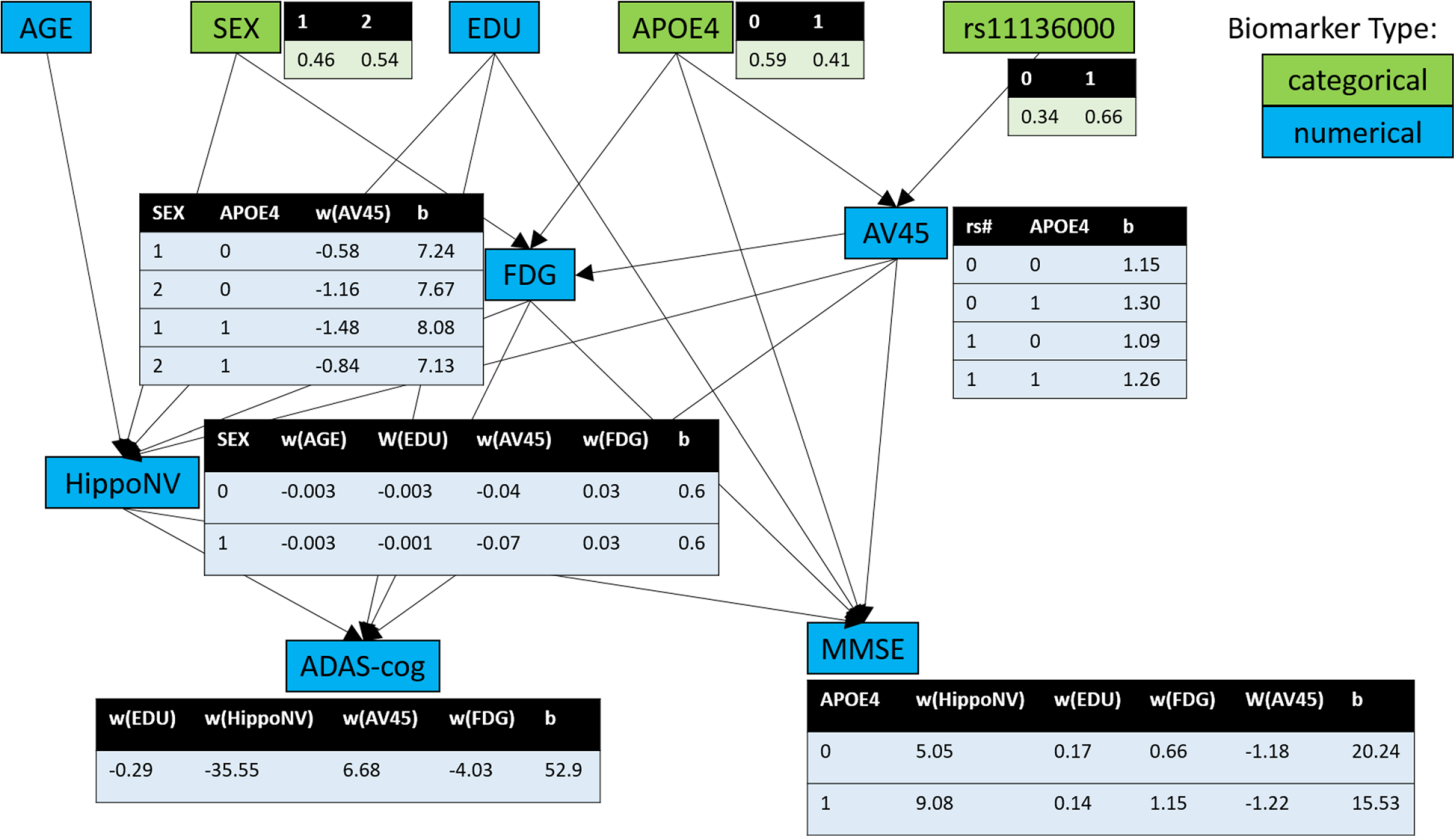
Learn mixed type Bayesian network using heterogeneous multimodality data at baseline

The final BN model is shown in Fig. 1. Note that some variables in Table 2 are not shown in Fig. 1. This indicates that the algorithm was not able to detect significant and robust relationship among these variables with others. We use green to represent categorical variables while using blue to represent numerical variables. The probability tables of categorical variables and the parameters of the conditional Gaussian distribution *w*,*b* for continuous variables are shown along the DAG structure as well. For example, node HippoNV in Fig. 1 has five parents: sex is binary when the other four are numerical. The relationship between the HippoNV with other variables such as AGE, EDU, AV45, and FDG is characterized as a regression model, while parameters of this regression model vary according to the categorical variable SEX.

Overall, this network structure is consistent with the existing knowledge in AD literature. As expected [12–16], the APOE e4 was associated with higher amyloid burden (as measured by AV45 PET imaging) and lower cerebral glucose metabolism (as measured by FDG-PET). A direct impact of e4 to MMSE score was also identified in our results in agreement with previous reports [17, 18], although its underlying mechanism warrants further investigation. An association of the SNP rs11136000 with amyloid burden was also identified, in agreement with the potential role of clusterin (CLU, the gene that SNP rs11136000 is associated with) in A *β* clearance [19, 20]. Based on this study, it is also identified that there were direct relationships between amyloid burden and cognitive performance which may reflect the direct neurotoxic effect of A *β* and its derivatives or indirect impact through pathways that were not represented in the biomarkers we included in this study [21–23]. The direct interaction between cerebral glucose metabolism and cognitive function as identified in this study was also in agreement with prior knowledge [24–27]. The identified relationship between years of education and the cognitive performance might be a cognitive reserve effect as reported by a number of studies [28–31]. In summary, using Bayesian network, we identified inter-biomarker relationships that are in good agreement with the existing knowledge about AD.

### Evaluation of the prediction accuracy with BN

Besides comparing our results with AD literature, we further pursue numerical validation. Specifically, as “MMSE” and “ADAS-cog” are two important clinical outcomes, it is of interest to see if the learned BN owns significant prediction capability of the two outcomes. Thus, in this section, we compare the prediction capability of BN with three common regression techniques (implemented in R environment), such as linear regression (lm()), decision tree (rpart()), and random forest (randomForest()). The target metric we would like to measure and compare is mean square error (MSE), which serves as the goodness of fit in a regression problem. We use 10-fold cross validation to obtain unbiased estimates of MSE. To set up cross validation procedure, we randomly divide the original dataset into 10 subsamples. In each round, a single subsample is retained for testing the model while the remaining nine subsamples are used as training set.

Table 3 lists the mean and standard deviation of MSE of the models. In terms of the average of the MSE, the BN achieves a better accuracy than the linear regression and decision tree in both MMSE and ADAS-cog prediction, while its performance is close to the random forest which has been known to be a very powerful prediction model despite its black-box nature. Similar observation could also be made in terms of the variance of the MSE.

**Table 3:** Ten fold cross validation MSE result

	Mean (SD)	
	MMSE	ADAS-cog
Bayesian network	2.810 (0.441)	35.380 (3.244)
Linear regression	3.125 (0.439)	38.748 (4.364)
Decision tree	3.758 (0.552)	42.195 (4.306)
Random forest	2.914 (0.330)	35.218 (4.932)

### Validation of the identified BN via the covariance patterns

**Fig. 2:**
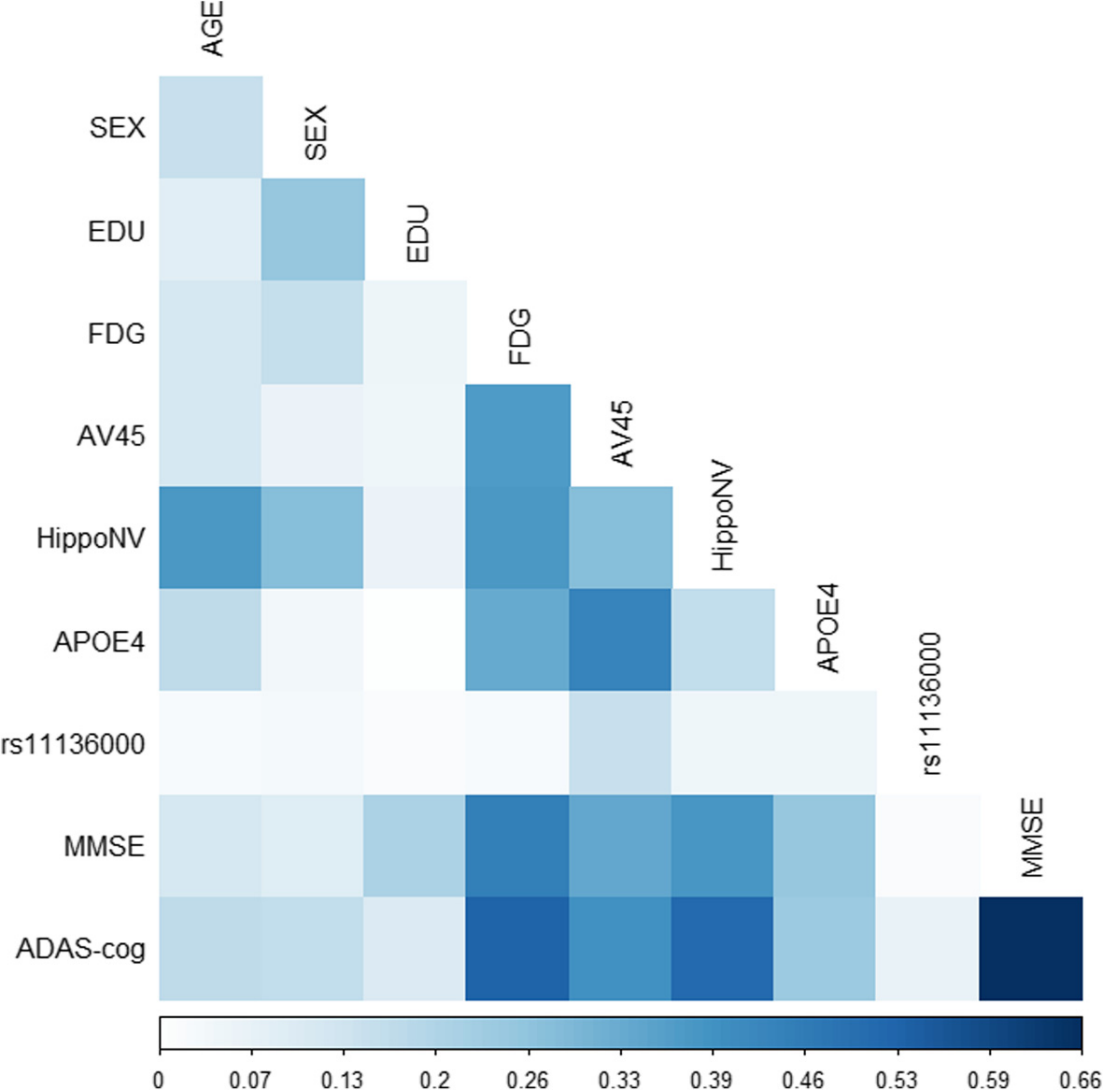
Visualization for heterogeneous correlation matrix

We also analyze the covariance patterns to help validate the learned BN model. The covariance patterns essentially characterize the undirected associations among variables. Thus, a BN model that aims to explain the influential relationships between the variables is expected to be able to explain the associations that are observed in data. Specifically, to derive the associations among variables, we use Pearson correlation for continuous variables, polychoric correlation for categorical variables, and polyserial correlation for a categorical variable and a continuous variable. The heterogeneous correlation matrix is computed using R package “polycor”. Figure 2 shows the associations we have observed from the biomarkers. Each row/column represents one biomarker. The color intensity shows the strength of an association. Note here that we only present the magnitude of the associations to focus the purpose on validation with the BN model. Overall, the association patterns revealed in Fig. 2 is consistent with our learned BN model. For instance, from Fig. 2, it is clear that the ADAS-cog is strongly associated with the variables FDG, AV45, HippoNV, and APOE4. While this is consistent with the BN as shown in Fig. 1, we also notice that in Fig. 2, we could not detect that the association between APOE4 with ADAS-cog could be mediated by the variable FDG. Thus, by learning the BN model, we could identify more layers in the relationships between the variables and could shed light to useful discoveries of the underlying mechanism of the disease progression.

### Validation of the identified BN via RuleFit

In order to validate the structure of the learned BN, another approach we propose to use is the RuleFit [32] method. RuleFit is a powerful method to discover complex interactions among variables. Again, it is a predictive model, so it lacks the capability of the BN to provide possible explanations of the relationships among the variables. But in the same spirit as the use of the association patterns to validate the BN model, we hope to see consistence between the BN structure with the interaction patterns the RuleFit could identify.

Thus, we apply the Rulefit on our data to identify the interactions among the biomarkers that can predict the two outcomes, MMSE and ADAS-cog. Table 4 lists the five rules we have identified. Column 1 gives the scaled importance for each rule. Column 2 (support) refers to the fraction of the samples in the dataset to which the rule applies. Apparently, it seems that there is great consistence between the two methods. For example, to predict MMSE, both the BN and RuleFit identified that HippoNV, FDG, EDU, and APOE4 are important. And to predcit ADAS-cog, both the BN and RuleFit identified that FDG, HippoNV, and AV45 are important. There are some interesting differences as well, e.g., RuleFit identified that the interaction between AGE and HippoNV is important to predict MMSE; however, it is revealed in the BN model that HippoNV actually mediates the effect from AGE to MMSE. Thus, given the consistency in the results, we could conclude that the BN model can provide more details of the underlying relationships among the variables.

**Table 4:** RuleFit: 10 most important rules

Impo.	Supp.	Rule
y: MMSE		
100	0.78	61.85 <*AGE*<86.85 and HippoNV >0.38
91.3	0.81	AGE <85.75 and FDG >5.78
74.6	0.15	FDG <5.85 and AV45 >1.11
68.2	0.06	EDU <19.5 and HippoNV <0.38 and APOE4=1
46.9	0.75	5.76 <*FDG*<7.25
y: ADAS-cog		
100	0.73	FDG >5.75 and HippoNV >0.39
62.5	0.65	FDG >4.9 and 1.02 <AV45 <1.51
41.2	0.72	EDU <19.5 and HippoNV <0.55
41	0.44	FDG >6.34 and rs3764650=0
35.9	0.17	1.23 <AV45 <1.63 and rs744373=0

## Conclusions

In this paper, we propose to use the mixed type Bayesian network to model the interactions among heterogeneous multimodal biomarkers. We conduct this study using ADNI baseline dataset and find that the learned BN model provides findings that are consistent with the AD literature. We further validate the learned BN structure via the prediction accuracy of clinical outcomes, capability to explain association patterns among variables, and comparison with powerful feature selection method. In future work, we would like to investigate the use of dynamic BN models to incorporate the temporal data that is available in the ADNI dataset. Critical changes of the biomarkers that may indicate disease progression may be discovered, and how these significant clinical events could be synthesized to be a systematical disease model is a very interesting and exciting research direction. Also, note that mixed type BN has been a challenging topic which is worthy of further methodological study. For example, different from the approach we used in this study, it is suggested to convert the continuous variables into discrete variables to enable application of discrete BN learning algorithms. Examples of these methods could be found in [8, 10]. Another approach is to directly model the mixed type variables, as the one we have used in our study. Examples of these methods could be found in [9]. The approach we used, although it has been a benchmark method and well evaluated in some applications, limits its applications to where only discrete variables could be parents of continuous variables. Both methods could lead to discovery of different types of relationships among variables, and different applications may require different approaches for optimal performance. Thus, we believe it will also be a future research direction.
